# Dissecting host-associated communities with DNA barcodes

**DOI:** 10.1098/rstb.2015.0328

**Published:** 2016-09-05

**Authors:** Christopher C. M. Baker, Leonora S. Bittleston, Jon G. Sanders, Naomi E. Pierce

**Affiliations:** Department of Organismic and Evolutionary Biology, Harvard University, Cambridge, MA 02138, USA

**Keywords:** *Nepenthes*, *Vachellia drepanolobium*, *Cephalotes*, DNA barcoding, metabarcoding, species interactions

## Abstract

DNA barcoding and metabarcoding methods have been invaluable in the study of interactions between host organisms and their symbiotic communities. Barcodes can help identify individual symbionts that are difficult to distinguish using morphological characters, and provide a way to classify undescribed species. Entire symbiont communities can be characterized rapidly using barcoding and especially metabarcoding methods, which is often crucial for isolating ecological signal from the substantial variation among individual hosts. Furthermore, barcodes allow the evolutionary histories of symbionts and their hosts to be assessed simultaneously and in reference to one another. Here, we describe three projects illustrating the utility of barcodes for studying symbiotic interactions: first, we consider communities of arthropods found in the ant-occupied domatia of the East African ant-plant *Vachellia* (*Acacia*) *drepanolobium*; second, we examine communities of arthropod and protozoan inquilines in three species of *Nepenthes* pitcher plant in South East Asia; third, we investigate communities of gut bacteria of South American ants in the genus *Cephalotes*. Advances in sequencing and computation, and greater database connectivity, will continue to expand the utility of barcoding methods for the study of species interactions, especially if barcoding can be approached flexibly by making use of alternative genetic loci, metagenomes and whole-genome data.

This article is part of the themed issue ‘From DNA barcodes to biomes’.

## 1. Introduction

In many species interactions, a host organism associates with a community of symbionts. Bacteria and protozoa in guts of lower termites, for example, help their hosts obtain nutrition from digestion-resistant foods [1]. Some 300 species of insects and mites have been found accompanying colonies of *Eciton burchellii* army ants and are known to depend at least in part on the ants [2]. Lichens, themselves symbioses of fungi and algae or cyanobacteria, host distinctive communities of bacteria on their surfaces, including lineages known almost exclusively from lichens [3]. These kinds of interactions are distinguished from simpler host–symbiont relationships by the potential for interactions among symbionts, and from studies of communities in abiotic contexts by the role of selection and phylogeny in shaping host interactions with symbionts. While species associations such as these have long been studied with a variety of approaches, DNA barcoding methods have in recent times become a useful addition to researchers' toolkits.

Barcoding can help classify symbiont taxa that would otherwise be difficult to identify. For many symbionts, morphological characters are inconspicuous or insufficient for identification, and for these organisms DNA identification may be helpful. Insect juveniles, such as those associated with ant colonies, often have few good identifying characters; bacteria and fungi likewise can be hard to identify. In these cases, DNA identification may help reduce the time and effort for identification, or may help identify cryptic species (e.g. [4–6]). Even where species are undescribed or are not included in sequence databases, similarity-based clustering of DNA barcodes allows organisms to be placed into groups that may be treated like species; such groups are often referred to as ‘operational taxonomic units' or OTUs [7]. Since only small quantities of DNA are required, barcoding methods in general have broad application for species identification—they do not necessarily require intact specimens and can therefore be used with samples ranging from soil for biodiversity assessment [8], to fecal samples for diet analysis [9,10], and even previously parasitized leaf samples for the identification of emerged leaf miners and their parasitoids [11].

Using DNA barcodes can also provide insight into organization at the level of the whole community, by facilitating the rapid profiling of entire symbiont communities. Symbiont taxa often vary considerably among individual hosts, as well as between different host taxa or habitats, and parsing this variation requires analysis of the symbiont communities associated with many individual hosts. Of course this is not specific to mutualistic symbionts, and indeed barcoding has been used to good effect across a wide range of species interactions, such as assessing variation in parasitoid communities [12]. Furthermore, some patterns, such as interactions among the symbiont taxa themselves, may only be visible if the whole symbiont community is considered [13]. Community-level analysis has been especially pertinent to microbial symbioses, such as gut bacterial communities. In these cases, the combination of DNA barcoding with high-throughput sequencing technologies has facilitated the taxonomic profiling of complex communities through the simultaneous sequencing of many thousands of DNA barcodes from each sample, often termed ‘metabarcoding’.

DNA barcodes also permit the analysis of species interactions on evolutionary timescales. DNA barcodes are not just arbitrary species labels but, like any other part of the genome, contain the signature of their evolutionary past: recently diverged taxa tend to have more similar sequences than distantly related taxa. Using barcode data to compare evolutionary relationships among host taxa with those among symbiont taxa potentially provides a way to detect relevant patterns in those evolutionary histories, such as codiversification between hosts and symbionts.

In this paper, we review three DNA barcode-based studies we have performed that demonstrate the broad scope for using DNA barcodes to study species interactions. First, we outline our study of arthropods residing in the hollow, swollen thorns of the African ant-plant *Vachellia* (*Acacia*) *drepanolobium* based on cytochrome c oxidase I (COI) barcodes. Second, we describe our use of 18S metabarcoding to identify arthropods and arthropod-associated protozoa in *Nepenthes* pitcher plants. Third, we detail our use of 16S metabarcoding to explore codiversification of gut bacterial communities with their *Cephalotes* ant hosts. Our studies serve to illustrate the scope and flexibility of barcodes as analytical tools in the study of species interactions.

## 2. Myrmecophile communities in *Vachellia drepanolobium* ant plants

DNA barcoding has proven valuable for examining communities of arthropods residing in domatia of the ant-plant *Vachellia* (*Acacia*) *drepanolobium*.

**Figure 1.**
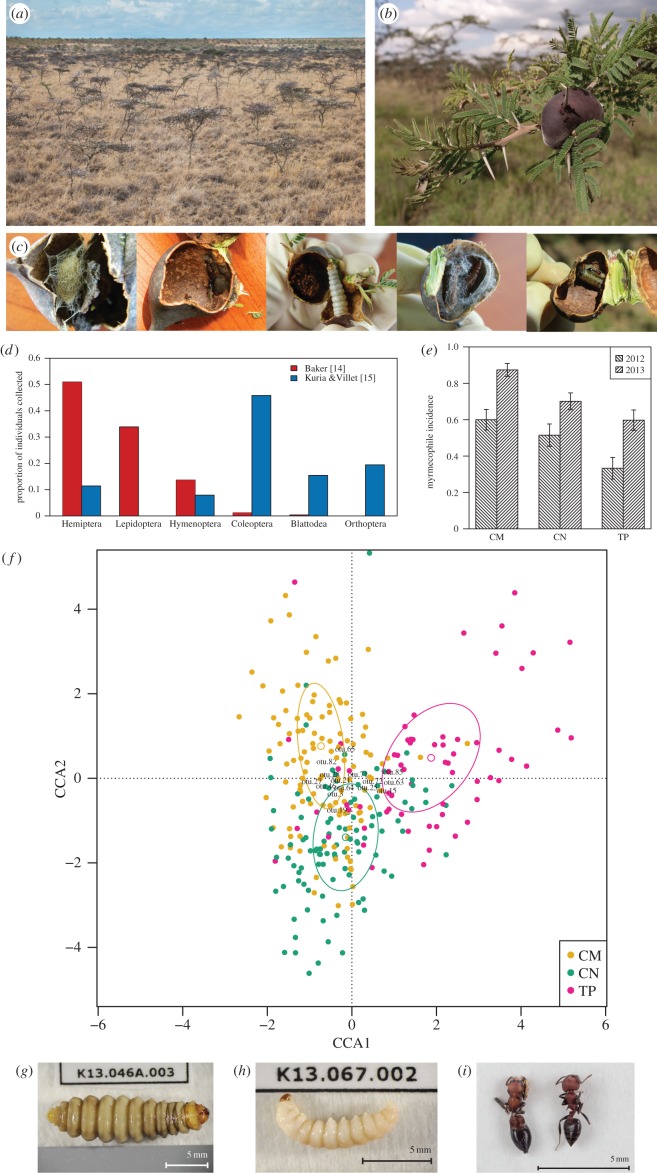
(*Overleaf.*) (*a*) *Vachellia drepanolobium* is typically the dominant tree species in East African black cotton savannahs—virtually all trees visible in the image are *V. drepanolobium*. (*b*) *Vachellia drepanolobium* is covered with stipular thorns to defend against large mammalian herbivores. Many of the thorns are swollen and hollow, and serve as domatia inhabited by mutualistic ants. (*c*) Many of the myrmecophiles in domatia of *V. drepanolobium* are immature forms that are difficult to identify using morphological characteristics. Molecular barcodes can be used to identify these myrmecophiles and link them to adult forms that are often better known or better described. Photo credit: Julianne Pelaez. (*d*) Domatium myrmecophile communities (red bars, from [14]) are dominated by Hemiptera and Lepidoptera, but these taxa are less common in canopy insect communities (blue bars, from [15]). Domatium myrmecophiles also include spiders and snails, but these are omitted here for consistency with [15]. Data from [15] are derived from table 1 of that paper under the Creative Commons BY 4.0 licence. (*e*) Trees of *V. drepanolobium* occupied by colonies of *C. mimosae* (CM) are more likely to host domatium myrmecophiles than trees occupied by colonies of *C. nigriceps* (CN), which in turn are more likely than trees occupied by colonies of *T. penzigi* (TP). From [14]. (*f*) Canonical correspondence analysis of myrmecophile communities showing that *C. mimosae* (CM), *C. nigriceps* (CN) and *T. penzigi* (TP) ants associate with distinctive communities of domatium-dwelling myrmecophiles. Plot shows the two canonical correspondence analysis axes (CCA1 and CCA2). Points represent individual trees and clearly separate according to the ant occupant, as denoted by colours of points. (*g*) The tortricid moth *Hystrichophora griseana* is found on trees occupied by *C. mimosae* and *C. nigriceps* ants, but not on those occupied by *T. penzigi*. (*h*) This gelechiid moth, *Dichomeris* sp., was found with all three ants. (*i*) This salticid spider, *Myrmarachne* sp. (left) is a convincing visual mimic of *C. mimosae* ants (right), yet surprisingly was found on trees occupied by all three ant species.

*Vachellia drepanolobium* is widespread throughout the East African tropics, often forming large mono-dominant stands in savannahs with hardpan grey soil or poorly drained black cotton soil (figure 1*a*) [16]. *Vachellia drepanolobium* is covered with hollow swollen-thorn domatia (figure 1*b*) that, at least on larger trees, are usually occupied by ants [16]. Three ant species nest obligately in these domatia: *Crematogaster mimosae*, *C. nigriceps* and *Tetraponera penzigi* [17]. A fourth species, *C. sjostedti*, also associates with *V. drepanolobium* trees but more commonly nests in trunk cavities created by cerambycid beetles or in the ground around the tree bases [18]. Each tree is normally occupied by a single ant species, but different trees, even within metres of one another, may be occupied by different species [18].

The obligate domatium-dwelling ants engage in a classic protection mutualism [19] with their hosts. In exchange for housing, as well as food in the form of extrafloral nectar, the ants protect their host plant from mammalian herbivores such as elephants, giraffe and antelope [20–22]. The ants vary, however, in the quality of their defence [17,23]. Among the three domatium-dwelling ants, the aggressive *C. mimosae* provides better defence than *C. nigriceps*, while *T. penzigi* does little to deter browsers [24]. And the ants impose other costs on their hosts: *C. nigriceps* prunes the plant's axillary buds, shaping growth and temporarily preventing flowering, while *T. penzigi* prunes the extrafloral nectaries, perhaps to reduce the risk of invasion by another ant colony [25,26].

The ants' effects are also evident in the diverse assemblage of other organisms found on the host plant. A 2012 study of insects in the tree canopy, using a morphospecies approach, found that canopy communities on trees occupied by *C. mimosae* and *C. nigriceps* were distinct from those on trees occupied by *C. sjostedti* and *T. penzigi* [15]. Other studies of specific tree inhabitants also describe preferential associations with ant species (e.g. [17,27]). Scale insects, for example, are found with *C. mimosae* and *C. sjostedti* [16], while neither *C. nigriceps* nor *T. penzigi* is typically found with scales. The lycaenid *Anthene usamba* specializes on trees occupied by *C. mimosae* [28]. The braconid wasp *Trigastrotheca laikipiensis* is a brood parasite of claustral colonies of *C. mimosae* and *C. nigriceps*, but is rarely found with *T. penzigi* [29], and *Acacidiplosis* gall midge parasites are found more frequently with *C. mimosae* ants than with *C. nigriceps* ants [30].

A wide range of myrmecophiles (ant lovers) is also found living in the domatia alongside the ants. The ant-occupied domatia constitute a highly unique habitat—heavily defended by the ants against intruders, environmentally stable and long-lived [31]. In response to this unique environment, we might expect domatium inhabitants in turn to be highly specialized. First, each of the domatium-dwelling ant species is highly aggressive, not only towards intruders that it detects, but also towards each other [18]. Myrmecophiles need to be able to avoid the ants' defences via mimicry, physical defences and/or engaging in mutualistic or manipulative interactions with the ants. We might therefore expect at least some myrmecophiles to specialize in their ant associations because of the degree of fine tuning required to interact successfully with their hosts. Second, we might expect some myrmecophiles to preferentially associate with one or more of the ant species if the ants differ in the benefits that they provide to the myrmecophiles, such as defence from predators. And third, we should see selection for lifestyles that capitalize on the stable and long-lived environment—for example, ant parasites with low costs and ant mutualists with low benefits [31]—and we thus expect domatium myrmecophile communities to be distinct from communities residing or transiently present in the canopies of the trees.

To explore the make-up of the domatium myrmecophile communities, we collected myrmecophiles exhaustively from 480 trees over 2 years at two sites in Kenya, for a total of 2361 individual myrmecophiles (see [14] for collection details). But deriving quantitative data from collections of domatium myrmecophiles is challenging. Many species are undescribed, and many of the myrmecophiles are immature forms that are often poorly known and difficult to identify (figure 1*c*). For example, out of the almost 600 individual Lepidoptera in our collection, 72.6% were larvae, 26.0% were pupae and just 1.4% were adults. DNA barcoding methods were therefore invaluable in examining these domatium myrmecophile communities, by serving in place of detailed morphological identifications [32].

We therefore sequenced COI barcodes for 1091 of our 2361 specimens in order to identify them. Since species-level taxonomic identifications were not always possible, we defined OTUs for these specimens using the uclust clustering algorithm [33]. We classified a further 28 specimens based on visual inspection where we failed to obtain good sequence. We also classified 1270 specimens that we did not sequence. These specimens belonged to six morphotypes, found with high abundance on a relatively small number of trees, for which standard COI barcode primers did not amplify (873 scale insects; see [34], but also see [35] for alternative primers) or for which the cost of sequencing all specimens did not appear to be justified (149 snails, 53 thrips and 132 ants belonging to three taxa). The OTU-based classification of most specimens was not sensitive to the type of clustering algorithm or choice of similarity threshold. Nonetheless, for a small number of specimens, clustering choices did affect whether those specimens were grouped with others or classified as separate taxa, and we regard those specimens as good candidates for future investigation using molecular or morphological methods.

Our myrmecophile collections revealed that domatium communities were indeed taxonomically distinct from canopy communities (figure 1*d* and [14]). Domatium communities were dominated by Hemiptera and Lepidoptera, but these were less common in canopy insect communities, which were dominated by Coleoptera. (Domatium myrmecophiles also included many spiders and snails, but these were not reported for canopy communities in [15]).

As with the canopy insect communities [15], the abundance of domatium myrmecophiles differed among the ant species. Among the three domatium-dwelling ants, *C. mimosae* was more likely to host myrmecophiles than *C. nigriceps*, which in turn was more likely than *T. penzigi* (figure 1*e* and [14]; see also [36]). Since most of the domatium myrmecophiles—particularly the Lepidoptera—are herbivorous [14], this pattern stands in contrast to the ants' defence against mammalian herbivores: *C. mimosae* is usually considered the best defender against large mammals, and *T. penzigi* the least effective.

Domatium myrmecophile communities also differed in composition among the ants. *C. mimosae*, *C. nigriceps* and *T. penzigi* ants tended to associate with distinctive communities of domatium-dwelling myrmecophiles (figure 1*f*), though communities varied widely within each ant species. Some myrmecophiles showed strong specialization, as expected. Scale insects, for example, were almost always associated with *C. mimosae* ants. The tortricid moth *Hystrichophora griseana* (figure 1*g*) was very common with *C. mimosae* and *C. nigriceps*, but almost never found with *T. penzigi*. But for the most part, we found limited evidence for strong specialization on ant species. In many cases, this was because the number of individuals from an OTU was too small to clearly establish ant specialization. But there were also many cases where abundant taxa appeared to show no particular ant association. For example, notwithstanding strong specialization of *H. griseana*, many Lepidoptera (e.g. *Dichomeris* sp. in figure 1*h*) were associated with all three ants, and the use of DNA barcodes helps rule out the possibility that these are really cryptic species.

Perhaps the most surprising finding of generalist ant association was in the case of the abundant *Myrmarachne* sp. salticid spiders (figure 1*i*). Despite extremely strong visual mimicry of *C. mimosae* (*C. nigriceps* and *T. penzigi* ants differ in coloration, and *T. penzigi* also differs in body shape), these spiders were not found any more commonly with *C. mimosae* than with the other domatium-dwelling ants. The visual mimicry probably plays no role in disguising spiders from the tree's ant residents, since most ants rely primarily on pheromones rather than visual cues to detect intruders. Instead, it probably serves to avoid predation by birds or parasitism by wasps. The close mimicry of *C. mimosae* combined with the fact that the spiders were found with ants other than *C. mimosae* suggest that selection favours mimicry of *C. mimosae* over other species of resident ant, presumably because they are the most bellicose species, and that predators are deceived by the spiders' appearance as a worker of *C. mimosae*, but do not attend to the mismatch between the spiders and the tree's resident ants.

Our ongoing study of domatium myrmecophile communities has benefited greatly from DNA barcoding. The use of barcodes allows myrmecophiles to be collected and preserved rapidly in the field; identification of specimens across a broad taxonomic range can then easily be performed later by non-specialists. Although species-level taxonomic identifications are not always possible, especially in taxa that are not yet well represented in sequence databases, community-level patterns can still be analysed by making use of similarity-based clustering into OTUs. Flexible, efficient and cost-effective molecular protocols allow good throughput and thus increase feasibility for medium- to large-sized barcoding projects, in turn facilitating the detection of community-level patterns (e.g. automation-friendly DNA extractions [37]; we have also had good results with phenol-chloroform extractions on an AutoGenprep 965 robot, and with basic Chelex bead extractions [38]). While data management can be challenging for larger projects, we have found well-designed sequence processing pipelines (e.g. the Barcode of Life Data System [39]) and open-source relational database applications (e.g. VoSeq [40]) to be useful for managing sequences and other associated data.

## 3. Inquiline communities in *Nepenthes* pitchers

The aquatic pools enclosed by leaves of carnivorous pitcher plants contain communities of arthropods and microbes, and have been used for decades to study community dynamics [41–43]. Like the poorly known inhabitants of ant domatia, the protists and small organisms living in these pitchers can be difficult to identify by morphological methods alone. Metabarcoding, also known as next-generation amplicon sequencing [8,44], is distinguished from conventional barcoding by operating on the collective DNA of the environment rather than the isolated DNA of individual organisms. Thus, metabarcoding can provide a broader and less biased view of the organisms living within pitcher communities.

**Figure 2.**
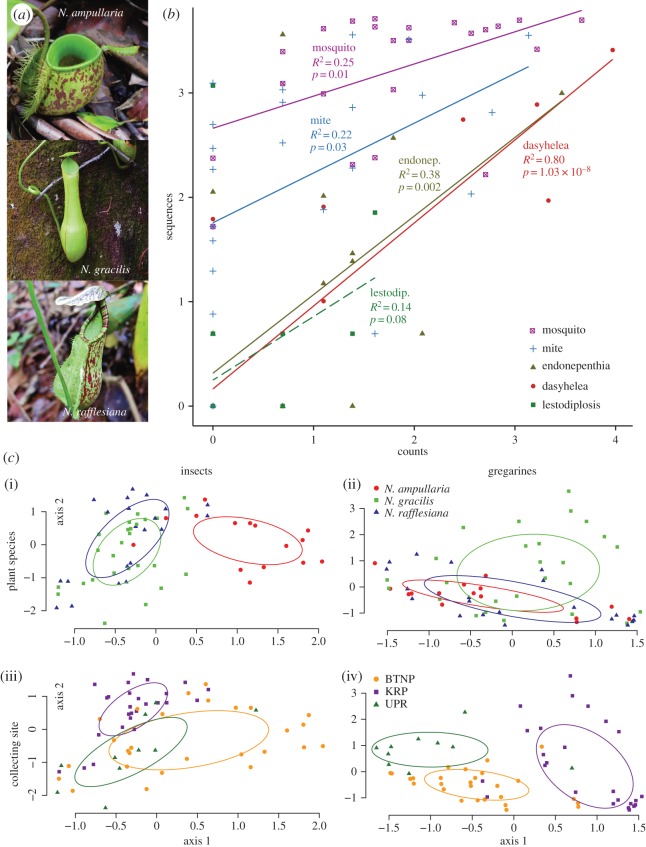
(*a*) Three species of *Nepenthes* pitcher plants studied in Bittleston *et al*. [46]. (*b*) Scatter plot of inquiline individual counts and 18S sequence counts plotted on a log_10_–log_10_ scale. Regression lines and *p*-values from the permutational linear models overlie the scatter plot. Solid lines are significant at *α* = 0.05. Reproduced from figure 3*a* of Bittleston *et al*. [46], copyright © 2015 by John Wiley Sons, Inc. Reprinted by permission of John Wiley & Sons, Inc. (*c*) Non-metric multidimensional scaling ordinations of insect communities ((i),(iii)) and gregarine communities ((ii),(iv)). Each point is a different pitcher plant, coloured by pitcher plant species ((i),(ii)) and by collecting site ((iii),(iv)). Ellipsoids are standard deviations of the points around the centres. Variation among insect communities is dominated by the difference between pitchers of *N. ampullaria* and pitchers of *N. gracilis* and *N. rafflesiana*. *Nepenthes ampullaria* is hypothesized to be more detritivorous than the other two species, which are predominantly carnivorous [47]. Variation among gregarine communities is dominated by variation among collecting sites. BTNP, Bukit Timah Nature Preserve; KRP, Kent Ridge Park; UPR, Upper Peirce Reservoir Park.

Pitchers of plants in the genus *Nepenthes* in Southeast Asia attract prey with extrafloral nectar, and have slippery edges and inner walls that cause insect visitors to fall in and drown [45]. The fluid inside the pitchers contains a mixture of rainwater and plant secretions. Pitchers catch and digest insect prey, but they also host thriving communities of aquatic arthropods, protozoa, bacteria and fungi, called ‘inquilines’ [41]. Some inquilines have only been found living in *Nepenthes* pitchers and appear to be endemic to these habitats [42]. To fully characterize and understand the communities within pitcher systems, we need a relatively unbiased view of the organisms present. Most previous studies of *Nepenthes* inquilines have been morphological [41–43], but in our recent study, we used metabarcoding to examine the eukaryotic communities within three *Nepenthes* (figure 2*a*) species in Singapore [46].

An important question with metabarcoding is how relative abundances of sequences compare to actual organism counts, and whether community structure can be recovered [48]. Since *Nepenthes* pitchers are relatively self-contained, whole organism counts of insects can be compared to metabarcoding OTU counts. In our study, we therefore compared counts of inquiline insect larvae with metabarcoded 18S rDNA sequences from the same samples, to see how well the metabarcoding captured abundances of these organisms [46]. Positive correlations were found between the counts and sequences (figure 2*b*), suggesting that metabarcoding can be useful for understanding the community structure of these organisms.

Our metabarcoding of *Nepenthes* pitchers also uncovered the presence of abundant gregarines (apicomplexan protozoa), which are obligate parasites of invertebrates [46,49]. The subclass Gregarinasina was the fourth most abundant eukaryotic group in *Nepenthes* pitchers, after insects, arachnids and algae. Mosquito larvae have been shown to ingest gregarine oocytes while feeding [50]. The gregarines then complete their lifecycle in the mosquito midgut, and new oocysts are released into the environment during defecation, emergence into the adult form, or upon ovoposition [50]. Morphological identification of gregarines in *Nepenthes* pitchers would be difficult, as they are small and can be hidden within the intestines of their hosts.

In our 18S metabarcoding study, insect OTUs largely mapped to dipteran inquilines living within the pitchers [46]. A bipartite network of insect and mite OTUs from the three *Nepenthes* plant species showed that these inquilines were significantly specialized. Certain OTUs tended to be found only within one plant species, while others were generalists found equally in all three hosts [46]. Insects adapted to the *Nepenthes* pitcher habitat might be able to distinguish between plant species, and adults might preferentially lay their eggs in certain species. Alternatively, conditions within the pitchers of different species may allow certain inquilines to thrive while inhibiting the growth of others.

Because gregarines are obligate parasites, and both insect and gregarine OTUs were in high abundances in pitcher habitats, we hypothesized that insect and gregarine diversity would follow similar patterns. To investigate this hypothesis, we performed a new analysis on the data from our previous publication [46] by separating the eukaryotic OTU table 1 (in paper [46]) into insect and gregarine tables, and rarefying those tables to 1922 and 200 sequences, respectively. Twenty-one samples had fewer than 200 gregarine sequences and these samples were removed from both the insect and gregarine tables. We used distance-based redundancy analysis (dbRDA, function *capscale* in R) with Bray–Curtis distances to determine the effects of plant species, collection site and collection year on insect and gregarine communities. On the dbRDA results, we used an ANOVA-like permutation test (function *anova* in the *vegan* package in R) with separate significance tests for each marginal term (plant species, collecting site or collecting year) in a model with all other terms. Contrary to our expectations, insect and gregarine communities exhibited different drivers of diversity (figure 2*c* and table 1). For both taxa, the majority of the variation was unexplained; however, a larger portion of the variation in insect community structure was explained by plant species (*p* < 0.001), while a larger portion of the variation in gregarine communities was explained by collecting site (*p* < 0.001). For both insects and gregarines, collection year did not significantly influence community structure.

**Table 1.** Results from distance-based redundancy analysis of *Nepenthes* insect communities (left) and gregarine communities (right) using Bray–Curtis distances, with host plant species, collection site and collection year as predictors. Insect and gregarine communities had different correlates of diversity: plant species was a significant predictor for insect communities, while collection site was a significant predictor for gregarine communities.

term	insects			gregarines		
	variance	*F*	*p*-value	variance	*F*	*p*-value
plant species	1.2055	3.3118	<0.001	0.8344	1.3719	0.108
collection site	0.585	1.6071	0.069	2.8718	4.7217	<0.001
collection year	0.1438	0.7899	0.587	0.4874	1.6026	0.086
residual	10.3738			17.3341		

Adult inquiline insects most likely can determine which plant host they are visiting when laying eggs, and we see that certain inquilines prefer certain plant species. The plant species-associated variation in insect communities seen in our new analysis primarily reflects a distinctive community in *N. ampullaria* relative to *N. gracilis* and *N. rafflesiana* (figure 2*c*). *Nepenthes ampullaria* is hypothesized to be less reliant on carnivory and more of a detritivore than other *Nepenthes* species [47]. The different ecology of *N. ampullaria* is potentially reflected in altered pitcher conditions, in turn selecting for different inquiline inhabitants.

Gregarine distributions, on the other hand, were better predicted by collection site than by host plant species. In our new analysis, the Kent Ridge Park (KRP) samples had gregarine communities that were more different than those from the other two sites, and geographically KRP is also further away. Gregarine parasites could have been introduced into the pitchers via adult inquilines, via prey species, or perhaps via abiotic vehicles such as raindrops. Considering the differences in diversity patterns of gregarines versus insects, we hypothesize that introduction via adult inquiline insects during oviposition is unlikely, as we would then have expected their distributions to be correlated. It is possible that gregarines could be encysted in a dormant stage within the pitcher fluid where they could use the assembly of many insects in one location to opportunistically infect new hosts. Alternatively, the gregarines may have complex infection and/or epidemiological dynamics with their host insects that we have yet to understand.

In general, insect inquilines appear to colonize *Nepenthes* pitchers more deterministically than gregarines, with certain organisms selecting specific host plant species, regardless of the location. Conversely, gregarines appear to colonize pitchers more stochastically, exhibiting a stronger correlation with collection site, an effect that could potentially be caused by some kind of dispersal limitation.

Metabarcoding provides a window into the complex interactions and patterns of biodiversity exhibited by pitcher plant systems. Barcode differences also help to discriminate between organisms (such as aquatic mites) that are often difficult to distinguish morphologically. Moreover, metabarcoding in this case has enabled us to identify microscopic gregarine parasites across multiple plant species and collection sites, and to uncover surprisingly different patterns of diversity between gregarines and insect inquilines. Barcodes are a valuable tool for generating and testing new hypothesis of community assembly, and can extend our investigations to organisms that are small and otherwise difficult to study.

## 4. Coevolutionary histories of animals and gut bacteria

The metabarcoding of host-associated microbial communities also has the potential to teach us something about the coevolutionary history of species relationships—interactions understood to be of major importance to a growing number of aspects of animal biology [51]. As with conventional barcoding of macrofauna, 16S rRNA gene-based barcoding of bacterial communities originated with the intent of identifying *which* taxa are present in a given environment. Since microbial taxonomy is still very incomplete [52], this typically involves the similarity-based clustering of 16S barcodes into OTUs, to uncover patterns revealed by the distribution of these ‘taxa’ across hosts.

Host animals, unlike abiotic environments, themselves have an evolutionary history. It is widely appreciated that the distribution of microbial OTUs among hosts is a reflection of (and, possibly, an influence on) that evolutionary history [53,54]: closely related animals frequently also host more similar microbial communities than do distant relatives [53,55–57]. But these patterns of correlation between host phylogeny and microbial community similarity could result from a range of processes. Microbes could be inherited across host generations, resulting in codiversification of microbial lineages as a consequence of diversification in their hosts. Alternatively, related hosts could simply provide similar habitats, filtering similar microbes from the environment. These different processes also imply differences in the strength and nature of the effects host and microbe can have on each others' evolution. Partly owing to this ambiguous mapping from community pattern to evolutionary process, the question of how to interpret phylogenetic correlation in animal microbiota remains controversial [58].

Some additional insight into the origins of these correlations can be gleaned from consideration of metabarcode sequences not simply as taxonomic markers, but explicitly in light of their own evolutionary relationships. Metabarcode sequences reflect a phylogenetic history that must be consistent with any proposed hypothesis for the origin of phylogenetic correlation, allowing us to place constraints on some of those hypotheses. For example, microbial diversification produced as a consequence of host diversification is constrained by the age of the host: consequently, the evolutionary distance between microbial barcodes in different hosts should have a recent upper bound if correlation between community similarity and host phylogeny arose via codiversification.

**Figure 3.**
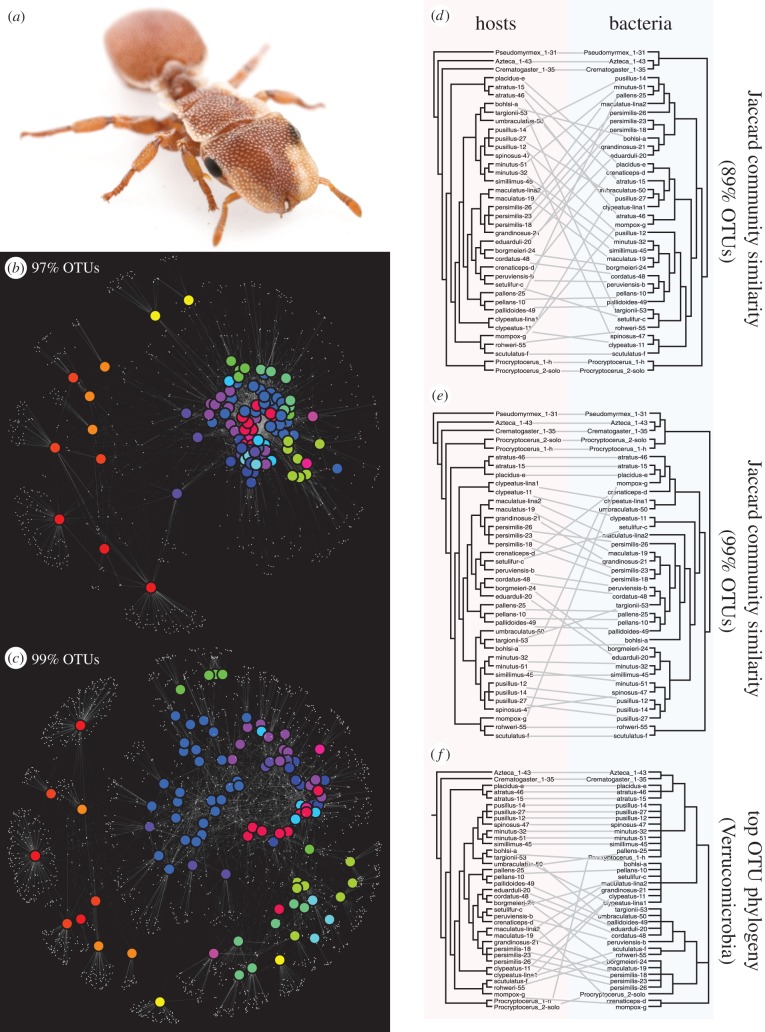
(*a*) *Cephalotes* ant and (*b*) 97% identity OTU network visualization of *Cephalotes* gut microbiota. Host samples (coloured icons) are connected by edges to 97% OTUs (small white dots). *Cephalotes* samples are coloured by host clade, with closely related species having similar colours. *Cephalotes* microbiota group together separately from non-*Cephalotes* microbiota (yellow, orange and red colours), as they share a large number of OTUs. (*c*) Identical to (*b*) except that OTUs are defined at 99% identity. Note that at 99% identity, far fewer OTUs are shared by all *Cephalotes* microbiota, but samples from related species continue to group together. (*d*) Tanglegram linking cladograms of host phylogenetic relationships with microbiota similarity relationships, defined by UPGMA clustering of Jaccard dissimilarities among *Cephalotes* colonies calculated with 89% OTUs. At this level, separation of *Cephalotes* microbiota from non-*Cephalotes* microbiota is retained, but phylogenetic relationships within *Cephalotes* are not reflected in microbiota similarities. (*e*) As in (*d*), except using 99% OTUs. At this level, similarity among microbiota also reflects phylogenetic relationships within *Cephalotes*. (*f*) Tanglegram linking phylogeny of the highest abundance member of the Verrucomicrobia from each *Cephalotes* colony to phylogeny of the hosts.

We can observe such a pattern in the gut microbial communities of South American turtle ants, in the genus *Cephalotes* (figure 3*a*). The diverse species of ants in this genus build their nests in empty cavities in trees and bushes, and host a dense gut microbiome that is thought to complement nutrient deficiencies in a largely herbivorous diet [59].

As has been reported in other systems, the gut microbiota of *Cephalotes* are correlated to host phylogeny (figure 3*b*). Using 454 metabarcoding of the bacterial 16S rRNA gene in guts from 25 *Cephalotes* species, we showed in a recent study that closely related ants also host more similar microbial communities [60]. But in the case of these ants, we were able to use the temporally structured evolutionary information within the barcodes themselves to give us some insight into how that similarity was likely to have arisen. Narrowing the similarity threshold used to define OTUs from the more typical 97% identity to 99% reveals the influence of more recent evolutionary history, splitting recently diverged microbial lineages that would have been collapsed into single OTUs at the wider threshold. Doing so increases the separation apparent between clades of related hosts in a network visualization of these communities (figure 3*c*). Wider OTU definitions also obscure correlations between clustering dendrograms of community similarity metrics and host phylogeny (figure 3*d*) that are apparent at narrower definitions (figure 3*e*). That such phylogenetic correlation is only apparent when considering information about relatively recent bacterial evolution is consistent with it being generated through processes like codiversification or phylogenetically restricted host shifts [61].

If codiversification does explain the similarity of communities from related host species, we should also be able to see a signal of host phylogeny in metabarcode sequences from individual microbial lineages. At least to some extent, we do (figure 3*f*). Taking advantage of the structure of diversity in the *Cephalotes* gut, we performed an additional analysis of our metabarcode data from [60] to examine a lineage of Verrucomicrobia that is both universally present and abundant in these communities, and for which there is usually only one dominant strain per host community. We took the representative 16S metabarcode sequence for the 99% OTU assigned to the Verrucomicrobia lineage that was most abundant in each *Cephalotes* colony, aligned all extracted sequences using MUSCLE and then built a pseudo-maximum-likelihood phylogeny of these barcodes using FastTree. A tanglegram analysis of this bacterial tree shows substantial but imperfect correlation with host phylogeny, suggesting that this lineage may indeed be codiversifying with the host. That this correlation is weaker than the aggregate signal for the entire community (figure 3*e*) further suggests that other lineages in the community are undergoing similar processes.

In principle, such lineage-by-lineage analyses offer the potential to sift through whole communities to identify the specific microbes shaping phylogenetic correlation in microbiomes—giving us a potentially powerful tool for understanding these complex systems. Separating lineages by their evolutionary fidelity to hosts could help to identify microbes especially likely to be of functional import, whether owing to explicit reciprocal coevolution with the host or simply as a by-product of having been a constant element of the host's internal environment.

In practice, limitations in typical metabarcoding approaches prevent drawing such conclusions with high sensitivity or specificity. The 16S rRNA gene evolves slowly. With the relatively short read lengths of current Illumina and Ion Torrent platforms, even tens of millions of years of divergence may only be supported by a handful of phylogenetically informative characters, resulting in poor phylogenetic reconstructions. Sequencing error further obscures this pattern.

Still, interrogation of the evolutionary history represented in metabarcode sequences has yielded a number of interesting cases, especially when combined with other techniques to increase the amount of useful information available for analysis. In bumblebees [62] and pyrrhocorid seed bugs [63], low-throughput follow-up sequencing of target lineages using specific primers permitted deeper exploration of trends observed in untargeted metabarcoding efforts. In vertebrates, techniques to reduce the impact of sequencing noise permitted the detection of patterns of host specificity from metabarcoding data, even though the underlying sequences were quite similar [55].

As new sequencing approaches are developed, analysis of evolutionary history directly from metabarcode data will become possible with more confidence. Long-read technology will allow the use of full-length gene sequences, provided current problems of read accuracy can be overcome. Even given current sequencing technology, changing the bacterial metabarcoding target to faster-evolving protein-coding genes will yield more phylogenetically informative information than the 16S gene. Recent work has already made this approach possible, either by the initial amplification of these genes [64] or by what is effectively post-hoc barcoding of microbial communities by sifting through shotgun metagenomic sequence data [65,66].

## 5. Conclusion

Our studies illustrate the value of DNA barcoding and metabarcoding for identifying taxa in host–symbiont community interactions. For organisms like myrmecophiles (often juvenile invertebrates) on *V. drepanolobium*, barcoding has provided us with a way to identify specimens that would otherwise be difficult to classify. Metabarcoding methods likewise have allowed us to detect and identify inquiline taxa in *Nepenthes* pitcher plants, and gut bacterial symbionts in *Cephalotes* ants.

But our studies also show how the utility of DNA barcodes can extend beyond the simple identification of individual symbionts, to the examination of ecological patterns [67]. This in part reflects the relatively high sample throughput permitted by barcoding methods, which facilitates the accurate profiling of entire communities, and offers the opportunity to assess interactions among symbionts and to identify patterns such as ecological convergence that may emerge only at the community level [68]. In our *V. drepanolobium* and *Nepenthes* studies, this high throughput was primarily realized through efficiencies in sample collection, sample processing and data analysis. As technology improves, an additional efficiency will become increasingly relevant: the availability of rapid in-the-field sequencing, using portable devices such as the Oxford Nanopore MinION [69], will permit almost real-time feedback on specimens and environmental samples. This will allow researchers to refine sample and data collection strategies on the fly (e.g. what are appropriate sample sizes and spatial scales for sampling?), and to generate new hypotheses that can be tested immediately instead of having to wait until the next field trip.

Although the value of DNA barcodes for species discovery and delineation has been challenged (e.g. [70]), we have found OTU clustering of arthropods and protozoa to be reasonably robust to choices of algorithm or parameters in both our *V. drepanolobium* and *Nepenthes* studies. Where results are sensitive to clustering choices, however, we are happy to adopt a relaxed approach to barcoding, and flag those specimens for further investigation using other markers or morphology, rather than rely solely on our barcoding data. Our analyses of differences in symbiont community composition between different host species largely sidestep uncertainty in the taxonomic placement or phylogenetic relationships of our OTUs: our analyses demonstrate community differences based on OTU abundances for each host, but not on the taxonomic labels attached to those OTUs, or on their phylogenetic placements (cf. [71]).

DNA barcodes can also provide a window on the evolutionary history of a host–symbiont association—a dynamic relationship shaped by selection and phylogenetic constraint that is absent in abiotic contexts. This reflects the fact that barcodes are not just taxonomic labels, but evolving DNA sequences that can be analysed for evidence of host–symbiont codiversification. In contrast to our *V. drepanolobium* and *Nepenthes* studies, the clustering of *Cephalotes* gut bacteria is sensitive to our choice of clustering threshold. But rather than being problematic, we are in fact able to use hierarchical clustering at different thresholds to our advantage, interpreting this sensitivity to parameters in light of expectations about the timescales of coevolutionary change.

As technology and methods improve, barcoding and metabarcoding approaches will become increasingly useful for ecological and evolutionary studies. Longer sequence reads and lower error rates, for example, will increase our capacity to draw inferences especially regarding recent phylogenetic history. The development of a wider range of sequencing targets will also help make barcoding approaches useful for a wider range of organisms and research questions. Indeed, as sequencing becomes cheaper, metagenomic datasets will allow appropriate barcode markers to be chosen *ex post* [65,66], or even for diversity assessments based on genome assemblies [72]. These approaches need not replace the simplicity of a single, standardized barcode region [32], but should nonetheless be embraced as a valuable expansion of the barcoding approach [73].

DNA barcodes will also become increasingly useful for ecological and evolutionary studies as sequence and other data accumulate in public databases. As these databases expand, we need to ensure that the widest possible selection of data can be accessed in an automated fashion, by encouraging researchers to annotate published data with as much machine-readable metadata as possible. Location, habitat or timestamp data on DNA barcodes, for example, may help generate more accurate pictures of species distributions over space and time, and the ecological correlates of those distributions. Conservation and barcode data can be combined in order to generate phylogenetically informed conservation assessments [74]. But barcodes and barcode-based taxon assignments also represent a natural and convenient way to connect a wide range of data from different datasets: images and information from museum and library digitization projects, location and other metadata from collections, morphological information, natural history observations, stable isotope data or even data on metabolic rates [75]. Combining datasets potentially allows researchers to uncover patterns across larger temporal, spatial or phylogenetic scales than would normally be feasible [67]. Combining multiple data types—e.g. on symbiont community composition, on genomic functional capacities and on the nature of trophic or other interactions among organisms—potentially allows us to, for example, identify emergent properties of communities or rules governing the assembly of symbiont communities [68], or to assess changes in community structure that might act as signals of ecological distress [76]. Connecting many disparate datasets so they are inter-referential is not a trivial challenge, but one that holds great potential for furthering our understanding of species interactions.
